# Religious Pro-Sociality? Experimental Evidence from a Sample of 766 Spaniards

**DOI:** 10.1371/journal.pone.0104685

**Published:** 2014-08-12

**Authors:** Pablo Brañas-Garza, Antonio M. Espín, Shoshana Neuman

**Affiliations:** 1 Middlesex University London, Business School, Hendon Campus, The Burroughs, London, United Kingdom; 2 Universidad de Granada, Departamento de Teoría e Historia Económica, Campus de Cartuja, Granada, Spain; 3 Bar-Ilan University, Department of Economics, Ramat-Gan, Israel; 4 CEPR, London, United Kingdom; 5 IZA, Bonn, Germany; University of Maribor, Slovenia

## Abstract

This study explores the relationship between several personal religion-related variables and social behaviour, using three paradigmatic economic games: the dictator (DG), ultimatum (UG), and trust (TG) games. A large carefully designed sample of the urban adult population in Granada (Spain) is employed (*N* = 766). From participants' decisions in these games we obtain measures of *altruism, bargaining behaviour and sense of fairness/equality, trust*, *and positive reciprocity*. Three dimensions of religiosity are examined: (*i*) religious denomination; (*ii*) intensity of religiosity, measured by active participation at church services; and (*iii*) conversion out into a different denomination than the one raised in. The major results are: (*i*) individuals with “no religion” made decisions closer to rational selfish behaviour in the DG and the UG compared to those who affiliate with a “standard” religious denomination; (*ii*) among Catholics, intensity of religiosity is the key variable that affects social behaviour insofar as religiously-active individuals are generally more pro-social than non-active ones; and (*iii*) the religion raised in seems to have no effect on pro-sociality, beyond the effect of the current measures of religiosity. Importantly, behaviour in the TG is not predicted by any of the religion-related variables we analyse. While the results partially support the notion of religious pro-sociality, on the other hand, they also highlight the importance of closely examining the multidimensional nature of both religiosity and pro-social behaviour.

## Introduction

Rules and norms of behaviour are fundamental elements of religions. Religions usually contain systems of ideas and rules about how life *should* be lived. The rules are not restricted to the family (or the individual) but cover also the social dimension, that is, how to behave in the community. These social norms prevent individuals from misconduct within the society (“*Thou shalt not kill*, *not commit adultery*, *not steal, not bear false witness against thy neighbour, …*”, Ten Commandments) and therefore restrain anti-social behaviour. Moreover, most religions promote generosity towards members of the society and also towards foreigners (in Islam the concept of *i'thar*, that is “preferring others to oneself”, especially towards those who need support; in Judaism, one is requested to give one tenth of his earnings to the needy; or “*One who does not give to the poor has no luck*”, Proverbs 28∶27). Religions also promote egalitarian distribution of resources. As noted in [1] and [2], egalitarianism is behind the idea of religious charity: sharing with those who have less.

One of the basic principles of religions is that God observes what humans do. It follows that individuals believe that they are constantly monitored by Him, who has the power to punish those who deviate from the norm, and reward those who follow the rules (e.g., [3], [4]). Punishment and reward are expected in both the current life and the afterlife. [5] provides empirical evidence (based on a large dataset, estimating equations of attendance of church services) that both the fear of divine punishment (Hell) and the expectation of divine reward (Heaven) significantly affect church attendance. Interestingly, belief in Heaven (reward) has a stronger incentive for church attendance than belief in Hell (punishment).

Accordingly, religiosity has proved to exert some effect on individual decision-making and behaviour. An extensive literature shows that religion and religiosity (as well as other cultural traits) matter to important economic phenomena, such as: educational attainments ([6], [7]); labour force participation ([8]); income and financial assets ([9]); marriage and inter-faith marriage ([10]); fertility ([8], [11], [12], [13], [14], [15], [16], [17]).

It was also demonstrated that religion and intensity of religiosity affect social interactions and attitudes: several studies relate to donations (e.g., [18], [19], [20]) and show that intensity of religious participation is positively associated with amounts donated in charity giving. In a similar vein, [21] finds a positive relationship between religiosity and trust in others and in institutions. [22] uses a sample of Latin American Catholics and show that religiously-active Catholics trust peers and institutions more, compared to non-active Catholics and individuals who belong to other denominations.

An accelerating phenomenon (in Europe and elsewhere) is the growing number of individuals who claim to have “no religion”. Data from the 2002–2010 waves of the European Social Survey (ESS) include 39.1% of respondents who identify their religion as “no religion” ([23]). A recent report published by the Pew Research Center's Forum on Religion & Public Life ([24]) claims that the third largest “religion” is the “no religion” – it is estimated that there are 1.1 billion individuals who claim to have “no religion” (16% of the world population; the majority lives in communist countries, 700 million in China). Christianity is the largest religion (2.2 billion individuals, comprising 32% of the world population) and Islam comes second (1.6 billion individuals, comprising 23% of world population). After the third “no religion” denomination, the fourth is Hindu (1 billion individuals) and the fifth is Buddhism (0.5 billion). Only 14 million individuals belong to the faith of Judaism (0.02 percent of world population). Given the pronounced share of the “no-religion” group it is essential to study their social behaviour as it will have significant effects on society.

Another significant phenomenon is the increasing influx of immigrants (into Europe and other regions), who compose a considerable share of the populations in many countries (see [23], Table 1). The intensified religiosity of immigrants (compared to natives) became a fundamental issue that could affect all spheres of life, including the economic and social domains. If religiosity is related to social behaviour as our study tries to explore (and the causal relation goes from religiosity to social behaviour), we can speculate that the outcome could be major changes in social behaviour and social institutions in Europe, which could as well affect other domains of the society and economy.

**Table 1:** Catholics versus non-Catholics.

	DG offer	UG offer	UG MAO	Trustor	Trustee
	(1)	(2)	(3)	(4)	(5)
*Catholic*	0.625	0.318	0.573**	0.026	−0.158
	(0.479)	(0.238)	(0.271)	(0.116)	(0.129)
*age*	0.024	−0.011	0.110**	0.007	−0.000
	(0.082)	(0.053)	(0.043)	(0.021)	(0.021)
*age sq.*	−0.000	0.000	−0.001**	−0.000	0.000
	(0.001)	(0.001)	(0.000)	(0.000)	(0.000)
*male*	−0.392	−0.055	−0.083	−0.082	0.043
	(0.392)	(0.214)	(0.242)	(0.103)	(0.111)
*house income*	−0.139	0.008	0.083	−0.002	−0.015
	(0.104)	(0.058)	(0.064)	(0.024)	(0.026)
*education*	0.008	0.049	−0.096	−0.015	−0.004
	(0.110)	(0.073)	(0.065)	(0.026)	(0.026)
*unemployed*	−0.467	−0.030	0.412	0.165	−0.100
	(0.417)	(0.240)	(0.290)	(0.103)	(0.128)
*married*	0.697	0.023	−1.239***	0.238	0.084
	(0.701)	(0.405)	(0.472)	(0.172)	(0.196)
*divorced*	2.030**	0.074	−1.043	0.058	0.231
	(0.800)	(0.699)	(0.734)	(0.275)	(0.294)
*widowed*	−0.398	0.061	−0.146	0.332	0.243
	(1.007)	(0.580)	(0.768)	(0.281)	(0.386)
*cohabiting*	−0.163	−0.849*	−0.308	0.450*	−0.301
	(1.216)	(0.463)	(0.721)	(0.259)	(0.310)
*impatience*	−0.096	−0.084**	0.093**	0.007	0.011
	(0.081)	(0.040)	(0.046)	(0.018)	(0.021)
*risk 1*	−0.238	0.011	1.174***	−0.134	−0.414***
	(0.562)	(0.309)	(0.371)	(0.133)	(0.150)
*risk 2*	0.653	−0.130	−0.169	0.183*	−0.099
	(0.403)	(0.269)	(0.316)	(0.104)	(0.104)
*risk 3*	1.695***	0.816**	−1.002*	0.920***	0.569***
	(0.598)	(0.371)	(0.596)	(0.219)	(0.185)
*cogn skills*	−0.148	−0.023	0.245**	0.001	0.096**
	(0.179)	(0.094)	(0.106)	(0.044)	(0.042)
*many immigr*	−0.302***	−0.104	0.027	−0.094***	−0.080***
	(0.098)	(0.065)	(0.060)	(0.027)	(0.025)
*big public sector*	0.421	0.027	0.298	−0.017	0.118
	(0.389)	(0.259)	(0.265)	(0.114)	(0.125)
*Constant*	9.834***	10.179***	3.351***	0.360	0.569
	(2.235)	(1.148)	(1.102)	(0.480)	(0.564)
*LR*	3.171***	1.520**	2.829***	95.259***	131.556***
*ll*	−2047.190	−1907.167	−2030.929	−436.102	−414.165
*N*	766	766	766	766	766

Notes: Dependent variables are displayed on top of the columns. Tobit estimates for models (1) and (2), OLS for model (3) and Probit for models (4) and (5). Robust SEs clustered by interviewer are presented (in parentheses). All regressions control for order effects.

* p<0.1,

** p<0.05,

*** p<0.01.

It should be noted that all the studies cited above on the relationship between religiosity and pro-social behaviour are based on self-reported survey data, rather than the direct observation of the individual's attitude and behaviour. Nonetheless, there are several experimental studies that investigate the role of religious denomination and intensity of religiosity on social behaviour, using economic games. Yet the results vary and are not conclusive. For instance, [25] – using a sample of 64 subjects, at the age of 50 and over – investigates whether religious denomination and intensity of attendance of church services are correlated with cooperation. They are using a repeated public good experiment, and find that neither denomination nor church-attendance significantly affect contributions to the public good. The experiment was replicated by [26] using a sample of 144 students, yielding similar insignificant effects, although contributions appear to increase with frequency of church attendance among subjects attending religious services. Moreover, [26] also find that church attendance does not have a significant effect on the outcomes of a trust game. Similarly, [27] does not find clear effects of individual-level religious variables on cooperation and trusting behaviour, in public good and trust game experiments, with 255 and 181 subjects, respectively. Using a large sample from three European countries, [28] does however find a weak positive effect of religiosity, measured by time devoted to religious associations, on the amounts passed by the “trustors” to the “trustees” in a trust game. In a similar vein, [29] reports a positive correlation between attendance at religious services and donations to charities, in an experiment with 168 subjects. [30], using a sample of 102 men, finds that religious students (preparing to enter the clergy in India) are more cooperative in a public good game and give more in a dictator game than non-religious ones.

In order to avoid the causality problems associated with studies that look at correlations, recent research has made use of religious priming in economic experiments. Using two samples, of 50 and 78 subjects, [31] finds that individuals who were assigned to a treatment with a scramble-sentence task aimed at priming religious concepts, were more generous in a dictator game (although, importantly, a similar effect was found when priming subjects with words related to secular moral institutions). However, in a similar experimental setup, using a larger sample of 304 subjects and a modified ultimatum game, [32] did not find a significant effect of religious priming on subjects' “altruistic punishment” of unfair behaviour, although a significant positive effect was found for those subjects who had previously donated to a religious organisation.

In a large experimental study (*N*>800), [33] explores the impact of religious identity – which was made salient by using a sentence-unscrambling task – on: contributions in a public good game; giving in a dictator game; risk aversion, time discounting and behaviour in a labour market task. Results are unclear: after religious priming, Protestants contribute more to the public good, Catholics contribute less and become less risk averse, while Jews reciprocate more in the labour market game. Also, they find no evidence that “religious identity salience” affects discount rates or purely altruistic generosity in the dictator game. Based on two experiments with 69 and 547 subjects, [34] analyses the effect of explicit religious primes on subjects' behaviour in a prisoner's dilemma game. It suggests a positive effect of religious primes on cooperation, at least among Christians. See [4], for a review of previous research on religious priming and a discussion on the origins and the evolutionary roots of religious pro-sociality, and [35], for a critical examination.

Thus, the results regarding how religion affects social behaviour in economic experiments have been far less conclusive than what one would expect according to the notion of religious pro-sociality. In this paper, we aim to add to this literature of *Experimental Economics of Religion* (see [36]). Using a large heterogeneous sample of 766 subjects, sampled from the urban adult population in Granada (Spain), we explore how individual religious variables correlate with social behaviour in three canonical economic games. Specifically, from participants' decisions in these games we obtained measures of *altruism* (giving in a dictator game, DG), *bargaining behaviour and sense of fairness/equality* (offer and minimum acceptable offer – MAO – in an ultimatum game, UG), *trust* (passing the money in a binary trust game, TG) and *positive reciprocity* (returning part of the trusted amount in the TG). See Methods. Note that the causality of the relationships we study can also run from pro-sociality to religiosity: e.g., it might be that people who share some social preferences are more likely to affiliate with a particular religious denomination. However, the very low share of respondents who declared having changed their religious denomination (for instance, *not even one individual* in our sample declared having been raised in a “non-religious denomination” and, later on, converted into a religious one; see below) suggests that causality runs mainly from religiosity to social behaviour, and not the other way around. Yet, although we include a large set of statistical controls in the analyses, there might exist unobserved third variables that could confound the relationships under investigation, so that causality problems cannot be fully ruled out and concerns regarding this issue may thus be valid.

Three dimensions of religiosity are considered and examined: the subjects' *religion/denomination* (61.6% are Catholics; 2% Muslims; 0.8% Evangelicals; 4.3% have other religions; and 31.3% are claiming to have “no-religion”, hereafter NR; we find a similar figure in Europe (39.1% NR, using ESS); frequency of *church-attendance*; and if the respondent *changed her/his religion* at some point in her/his life (from any denomination to another, including NR; for instance, 12.3% of respondents in the sample changed from Catholic to NR). The terms “no religion” and “non-believer” will be used interchangeably. See Methods.

Using the above information, this paper aims at answering the following questions: do Catholics (compared to the rest of the sample) exhibit a different social behaviour? Are those who claim to have no-religion (with respect to the rest of the sample, i.e., believers in any denomination) less or more pro-social? Is it just denomination that matters, or is religious intensity (measured by attendance to religious services) the key variable explaining social behaviour? And, finally, are religion-specific social values transmitted from parents to children? Data on religious conversion can help in answering the last question. It could be indicated by an examination of a group of individuals who currently share the same religion and comparing two within sub-groups: those who always had that religion, versus the sub-group that changed denomination (i.e., was raised within a different religion).

While these are interesting general questions, given the multidimensional nature of both social behaviour and religiosity, it is also essential to unravel in which specific dimensions are religiosity and social behaviour interconnected. Our set of experimental variables will facilitate such an examination.

We believe that our results provide a true reflection of the relationship between religiosity and social behaviour, and thus contribute significantly to the relatively scarce existing experimental literature. Our findings are trusted to be highly reliable due to (i) the use of several types of games: DG, UG, and TG; (ii) the length of the questionnaire (more than 100 items on a large variety of issues), which makes it unlikely that religious priming – related to having answered a few questions regarding religiosity – affects subjects' subsequent behaviour in the games; (iii) the large sample; (iv) the composition of the sample, that includes representative ordinary people, with varied socio-demographic characteristics, rather than only University students who compose the majority of samples in experimental economics studies; and (v) the unique sample that does not consist of only self-selected volunteers who come to the lab (which is common in most studies). Instead, interviewers went to the respondents' places. *The last two features are exclusive and innovative* and distinguish our experiments from the standard experiments presented in the literature. Although self-selected students (i.e. the typical subject pool of economic experiments) seem to have social preferences similar on average to those found in the general population ([37]), still more behavioural heterogeneity is expected in more representative samples (e.g., [38]). Heterogeneity in both religion-related variables and social behaviour are fundamental for our analyses.

The following section presents the findings, and the last section offers concluding remarks and implications. A detailed description of the variables and the procedures used can be found in Methods.

## Results

We will first explore *if the religion/denomination per se displays a significant relationship with social behaviour.* Two sub-populations are compared:

Catholics with the rest of the sample (regressions presented in Table 1), that is, the majority denomination vs. the rest;

NR with all others (including Catholics, Table 2), i.e., non-believers vs. believers.

**Table 2:** Non-believers/No religion versus believers.

	DG offer	UG offer	UG MAO	Trustor	Trustee
	(1)	(2)	(3)	(4)	(5)
*no-religion*	−0.939*	−0.547**	−0.645**	0.039	0.181
	(0.506)	(0.251)	(0.318)	(0.126)	(0.123)
*age*	0.024	−0.011	0.109**	0.006	0.000
	(0.083)	(0.053)	(0.043)	(0.021)	(0.021)
*age sq.*	−0.000	0.000	−0.001**	−0.000	0.000
	(0.001)	(0.001)	(0.000)	(0.000)	(0.000)
*male*	−0.401	−0.055	−0.110	−0.090	0.050
	(0.397)	(0.218)	(0.240)	(0.101)	(0.112)
*house income*	−0.139	0.007	0.086	−0.001	−0.016
	(0.104)	(0.058)	(0.064)	(0.024)	(0.026)
*education*	0.019	0.056	−0.090	−0.016	−0.005
	(0.110)	(0.073)	(0.064)	(0.027)	(0.026)
*unemployed*	−0.441	−0.015	0.428	0.164	−0.107
	(0.412)	(0.239)	(0.292)	(0.103)	(0.128)
*married*	0.638	−0.017	−1.265***	0.246	0.091
	(0.709)	(0.415)	(0.468)	(0.173)	(0.196)
*divorced*	1.974**	0.041	−1.061	0.068	0.241
	(0.800)	(0.701)	(0.739)	(0.274)	(0.293)
*widowed*	−0.434	0.038	−0.172	0.332	0.249
	(1.015)	(0.588)	(0.761)	(0.282)	(0.385)
*cohabiting*	−0.076	−0.786*	−0.285	0.434*	−0.312
	(1.210)	(0.472)	(0.734)	(0.261)	(0.307)
*impatience*	−0.097	−0.085**	0.094**	0.008	0.010
	(0.081)	(0.040)	(0.046)	(0.018)	(0.021)
*risk 1*	−0.200	0.036	1.201***	−0.137	−0.422***
	(0.555)	(0.304)	(0.376)	(0.134)	(0.150)
*risk 2*	0.673*	−0.120	−0.162	0.181*	−0.101
	(0.404)	(0.268)	(0.320)	(0.104)	(0.104)
*risk 3*	1.647***	0.780**	−1.008*	0.934***	0.572***
	(0.593)	(0.371)	(0.604)	(0.216)	(0.186)
*cogn skills*	−0.145	−0.022	0.249**	0.002	0.095**
	(0.179)	(0.095)	(0.106)	(0.044)	(0.042)
*many immigr*	−0.313***	−0.112*	0.027	−0.091***	−0.079***
	(0.098)	(0.068)	(0.059)	(0.027)	(0.026)
*big public sector*	0.415	0.021	0.298	−0.016	0.117
	(0.385)	(0.257)	(0.267)	(0.114)	(0.125)
*Constant*	10.481***	10.611***	3.847***	0.346	0.419
	(2.282)	(1.172)	(1.134)	(0.478)	(0.548)
*LR*	3.214***	1.612**	2.879***	93.804***	136.593***
*ll*	−2045.933	−1905.847	−2030.549	−436.072	−413.943
*N*	766	766	766	766	766

Notes: Dependent variables are displayed on top of the columns. Tobit estimates for models (1) and (2), OLS for model (3) and Probit for models (4) and (5). Robust SEs clustered by interviewer are presented (in parentheses). All regressions control for order effects.

* p<0.1,

** p<0.05,

*** p<0.01.

As in many other studies within the field of the *Economics of Religion*, “no-religion/not-believing” is also considered a religious denomination (see for instance, [39]). We do not relate specifically to social attributes of other religions (e.g., Evangelical, Muslim), due to their small sample sizes.

Five models are presented in each Table (columns (1)–(5)): DG and UG offers (in €, from 0 to 20) are the dependent variables in models (1) and (2), using Tobit regressions; column (3) explores UG MAO (in €, from 0 to 10), using an OLS regression model; finally, (4) and (5) are Probit models analysing behaviour in the roles of TG trustor and trustee, respectively. These same specifications were used in [37]. Alternative specifications yield qualitatively similar results. As in [37] robust standard errors are clustered on interviewers.

Socio-economic variables are included in order to arrive at net effects of our core variables, controlling for socio-economic differences between respondents. The same control variables are used in the two regression sets presented in Tables 1 and 2. Their effects are not much different in the two tables:

*Age* has an inverse U-shaped parabolic effect on the individuals' sense of fairness (UG MAO). Both *age* and *age-squared* are significant, indicating that MAO increases with age, reaches a maximum at about 55 and then decreases. No any other relevant effect is found to be related to *age*.

*Married* people are less prone to ask for equal shares (MAO) in the UG, indicating that they behave closer to the Nash equilibrium compared to singles. *Divorced* are more generous (DG). *Cohabiting* individuals offer less in the UG but trust more (pass the money as trustors) in the TG. However, both estimates are only marginally significant.

*Impatient* subjects offer less as proposers in the UG – they seem to be less strategically generous – but they ask for a larger share of the pie as responders. Obviously, impatient individuals are not easy to manage in bargaining and agreement processes. A deeper analysis of this result is reported in [40] where it is argued that impatience may be associated with a preference for spiteful competition in bargaining.

Turning to the effect of *risk attitudes*: risk-lovers in the gains' domain (*risk 1*) ask for more money in the UG (which is somehow a risky strategy) but they don't reciprocate in the TG (indicating that they are not very pro-social). Quite consistently, those who are ready to lose money (*risk 3*) risk their own money as trustors in the TG. Contrary to *Risk 1* these subjects seem pro-social: they share more in the DG and UG, ask less in the UG, and trust and reciprocate more in the TG. In any case, these results should be treated with caution, due to the use of hypothetical incentives for the elicitation of risk attitudes and given that the three *risk* variables are correlated (multicollinearity).

Individuals with better *cognitive skills* demand more money as responders in the UG, but they are also more prone to return (to reciprocate as trustees) in the TG, indicating that they may have a larger sense of reciprocity or, perhaps, of social responsibility.

Finally, those who claim that there are too *many immigrants* share less in the DG, which suggest that people who have little empathy for foreigners are also not so nice with locals – although it could be argued that they overestimate the likelihood that an immigrant will be the recipient of their offer. In addition, they offer less in the UG, they don't pass money in the TG, and also don't give the money back in the TG. Clearly, those who do not like immigrants are not very pro-social.

No significant effects of *education*, *income* or *gender* are found. We can therefore conclude that socio-demographics are not very relevant, but some specific personal characteristics related to preferences (risk attitudes, impatience) or cognitive skills are affecting decisions in several games.

Turning now to our core variable of *religious denomination*, Table 1 focuses on *Catholics* versus the rest of the sample, including NRs. We do not find any sound result rather than the positive relationship of *Catholic* with UG MAO. That is, Catholics tend to ask for more money as responders in the UG. Since we do not find any effect related to generosity (either pure (DG) or strategic (UG proposer)), trust (TG trustor), or reciprocation (TG trustee) we may say that there is a positive relationship between being Catholic and the aversion to disadvantageous, but not advantageous, inequality.

Interestingly, when the sample is restricted to “standard” religions only, excluding NRs, the effect of UG MAO becomes insignificant too (regression results not presented, can be provided upon request). We can therefore conclude that Catholics do not exhibit a different pro-social behaviour compared to members of other faiths.

In Table 2 the sub-sample of *NR*s is contrasted with the rest of participants (i.e., individuals who belong to the “standard” religions, including Catholics). Results are sharper now: those who classify themselves as NRs are less generous in the DG (although marginally), offer less as proposers in the UG and claim less money as responders (that might be indicative of a less strict sense of fairness). Hence we may conclude that NRs are less generous and not strongly driven by fairness/equality.

Interestingly, NRs are not different from “believers/individuals with a religion” in terms of trust: neither in terms of passing the pie to the second mover (*trustor*) nor in terms of returning the money (*trustee*). Given that previous results have been inconsistent (e.g., [26], [27], [28], [33]), and based on our carefully-designed large sample, we may conclude that the effects of believing in a religion on trust and trustworthiness, if any, are not clear and may be influenced by other factors, such as the country of residence, the specific religion or other institutional/contextual variables.

We will now relate to the relationship between *intensity of religiosity* (measured by frequent attendance of church services) and social behaviour, by distinguishing between active worshipers who go to church (place of worship) at least once a month and non-active ones who do not go to church on a regular basis (less than once a month). Table S1 in File S1 reports these regressions.

In order to hold constant the effect of denomination and focus on intensity of religious performance, we will relate to the sub-sample of Catholics, who constitute over 60% of the sample. All other religions have a very low representation that does not allow for a meaningful distinction between active and non-active worshipers (Muslims −2%, Evangelicals −0.8%, and all other religions combined −4.3%). NRs compose more than 30% of the sample, however a distinction between active- and non-active attenders of church services is obviously meaningless.

An examination of the effects of the control variables shows some differences between the whole sample and the subsample of Catholics. Nor *age*, neither the *marital status* of married are significant predictors of UG MAO, and cohabiting is no longer affecting TG behaviour. The effect of *impatience* disappears for Catholic respondents while the connection between risk attitudes and behaviour along the reported games remains basically unaltered. The effect of *cognitive skills* is also similar in the UG but its relationship with TG behaviour now relates to the role of the trustor and becomes negative. The *negative view about immigrants seems* to be less important for the subsample of Catholics, since its negative effect on pro-social behaviour is now restricted to the TG.

While we acknowledge that establishing causality may be even more problematic when studying religious participation, our conjecture is that frequent participation in church services will affect social/moral behaviour: the frequent attenders are more knowledgeable about religious texts and doctrines and in closer contact with the priest, inducing them to follow these moral rules and doctrines.

The performance of our core variable “being an *active* Catholic” is interesting: members of this sub-sample do give (marginally) more in the DG, which is reflecting a clearer sense of altruism and is quite consistent with what we saw in Table 1. In line with the results of [41] suggesting a negative relationship between ritual activity and MAO, we also find that *active* Catholics demand less money (than non-active Catholics) as responders in the UG.

To further explore these results, we performed regressions comparing active and non-active Catholics with NRs – i.e., excluding believers of other religions from the analyses – in terms of DG offers and UG MAOs (not reported; available upon request). We find that non-active Catholics do not offer significantly more than NRs in the DG (β = 0.478, n.s.) but active Catholics do (β = 1.543, p<0.05). With regards to MAO, non-active Catholics demand significantly more than NRs (β = 0.992, p<0.01) while active Catholics do not (β = 0.167, n.s.).

The difference between those with high and low attendance levels could reflect the effect of religious social interaction on social preferences (see [41]). While non-active Catholics have a more strict sense of self-centred fairness (i.e., they ask for a more egalitarian distribution as responders), active Catholics are playing closer to the Nash equilibrium (NE), accepting lower offers than non-active ones. Remember that the larger group of *all* Catholics (Table 1) exhibited a tendency of demanding *more* money. Combining the two seemingly contradictory findings leads to the conclusion that *within* the group of Catholics, there are major differences between active and non-active individuals. The larger sub-group of non-actives (67.8% of Catholics) dominates and leads to a larger MAO when no distinction (related to religious activity) is made.

Note therefore that while intensity of religious participation apparently strengthens the effect of being affiliated with a religion (versus a “no-religion”) on DG generosity, the former effect partially counteracts the latter when it comes to the rejection of unfair offers in the UG. It should be emphasised that behaving as if playing closer to the NE (in the case of active, compared to non-active, Catholics) as UG responder, is not necessarily an indication of more selfish behaviour. It is true that purely money-maximising subjects would accept any positive offer, setting MAO to its minimum value. However, it is also true that extremely pro-social subjects – very concerned with other players' payoffs – would accept any offer just to maximise the counterpart's profits (and social welfare). [42] presents support for this idea, using information from post-experimental interviews that shows that a large share of those who played the NE argued that “*maybe the other player needs the money*” as the principal reason to accept any offer, even zero. In the same vein, the results of [43] provide strong evidence that setting MAO to the minimum amount (i.e., zero) is a symptom of pro-social behaviour. In our case, the most obvious suggestion that playing the NE as responder does not indicate selfish behaviour is drawn from subjects' behaviour in the DG, which can be used to disentangle selfishness from pro-social preferences: the positive coefficient of *active* Catholics demonstrates that active Catholics give more money as dictators (column (1), Table S1 in file S1). This is clearly indicating that this sub-sample of *active* Catholics is *less* selfish. We may therefore conclude that active Catholics ask for less money in the UG (MAO) because they have a higher sense of generosity.

A different explanation of the MAO results might relate to the use of rejections as a form of “altruistic punishment” of norm violations. [44] shows that while religiosity generally *increases* the use of punishment of wrongdoing in a third-party setting, the specific belief in powerful, intervening Gods *reduces* it. It could be argued that church-attendance might be positively related with such belief in a “supernatural punisher”. Therefore, the lower MAOs shown by active, in comparison to non-active, Catholics could reflect a higher propensity to believe that it is not humans but God who should punish wrongdoers. However, the view of rejections in the UG as “altruistic punishment” of norm violations is being challenged on the basis that a large number of rejections seem to be triggered by pro-self, competitive motives (see [40], and the references quoted there).

Utilising the information on the third dimension of religiosity, namely *the experience of conversion into a religion that is different from the one educated/raised in* (see also [45] on converting-out), can shed light on the effects of childhood experience and cultural transmission from parents to their offspring. An extensive literature claims that values and norms (including religious norms) are transmitted across generations (e.g., [46], [47], [17]). The relatively large sample of NRs who were previously Catholic (94 out of 240 who are currently NRs were raised as Catholics and at some stage in life converted to NR), can be used to answer this interesting question.

Table S2 in File S1 presents the repeated regressions of DG offer, UG offer, UG MAO, trustor and trustee for the sub-sample of individuals who are currently NRs, including a dummy variable for the sub-group of subjects who were raised as Catholics before converting to NR.

The conclusion is quite straightforward. The two sub-groups of NRs are not different in terms of social preferences (insignificant coefficients in all five models). This result contrasts the theory that claims that values are transferred from parents to children. Our data does not lend support to this wide-spread theory. Therefore, perhaps the observed effects of religiosity on social behaviour have to do more with recently occurring life events than with early education. However, we should keep in mind that we relate to a distinct and very special (although growing) group that consists of NR individuals. Further research on this issue is warranted.

## Discussion

A large well-designed sample of Spanish individuals is used to explore the relationship between (i) religious denomination; (ii) religious intensity; and (iii) religious conversion and social behaviour, using the dictator, ultimatum and trust games.

The main results and implications of the paper are the following:

*i*) The sub-sample of “no religion” individuals (30% of the sample) is less generous compared to members of any “standard” religion, indicated by passing less money in both the dictator and the ultimatum games. In other words, those who classify themselves as NRs are more selfish. In addition, their MAO is lower, that is, they are more likely to accept unfair offers in the UG. Behaving *as if* playing the NE combined with selfishness in the DG is indicative of a perfect rational self-interested behaviour. Given the accelerating shares of “no religion” individuals in Europe (and elsewhere), and assuming that this result can be generalised for other places as well, we can project that the *society could become more self-interested as a result of the dominant role of non-believers.*

*ii*) Catholics are willing to reject unfair offers in the UG (higher MAO) more than the rest of the sample. They are not significantly different in terms of other social behaviours. In our Spanish sample the shares of “other religious denominations” is very low. More than 90% of the sample is composed of Catholics and “no religion” respondents. It follows that little can be proposed about the pro-sociality of other religions, and as a result this finding could not be generalised and applied to other (more religiously diverse) countries.

*iii*) Religious intensity (measured by active attendance of church services) matters above and beyond denomination: comparing religiously-active Catholics with non-active Catholics, we find that the former are more generous in the DG (while Catholics as a whole do not exhibit a differential behaviour in the DG) and claim less in the UG, that is, like in [41] MAO decreases with attendance. We can therefore conclude that there are differences in social behaviour *within* the group of Catholics, and *active Catholics exhibit a more pro-social behaviour than non-active Catholics* (similar results are shown in [29], and [30]). Due to the small shares of other denominations, it was not possible to distinguish between active- and non-active worshipers of other religions, other than Catholicism.

The two demographic phenomena described above: increasing numbers of “no religion” individuals on the one hand, and of actively-religious immigrants on the other hand, may have opposing effects on society. Given the much more pronounced growth rates of NRs, we arrive at quite pessimistic projections of a society that could become less generous and less pro-social. Unravelling the dynamic effects these two phenomena may have on societies' average social behaviour is an interesting issue for future research.

*iv*) It appears that only the current denomination (or “no denomination”) affects social behaviour. Respondents who were raised as Catholics and then converted to “no religion” do not exhibit different social preferences compared to “all life” NRs. While the “cultural transmission” literature ([47]) emphasizes childhood experiences and proposes that transmission of values/beliefs from parents to their offspring during childhood is affecting behaviour later in life, our results suggest that social behaviour is associated mainly with more recent, adulthood religious practice.

*v*) Like [26] and [27], we fail to find any significant relationship between religious denomination or religious activity and subjects' behaviour in either role of the TG. Given the large number of observations we analyse, such a systematic result is noteworthy and should be further examined. A potential explanation could be that trust games are not the proper device for the measurement of trust-related behaviour. Indeed, there is much debate on whether this type of problem should be interpreted in terms of trust and trustworthiness or instead in terms of an investment problem ([48], [49]).

## Methods

This section contains extensive information about the procedures and methods, divided in three parts. First, we describe the sample obtained through a stratified random method. Second, we focus on the protocol and the experimental games. The last section is devoted to the dataset and the large battery of controls it allows to employ (e.g., gender, income, education, age, political views, cognitive skills…).

### Sampling

The survey-experiment was conducted in Granada (Spain) in 2010. Detailed information of the protocol, including survey and experimental instructions, can be found in [37]. A stratified random method was used to obtain the sample. In particular, the city was divided into nine geographical districts, which served as sampling strata. Within each stratum, a proportional random method was applied to minimise sampling errors. This method ensures a geographically representative sample of the adult population of Granada.

The sample consists of individuals who agreed to complete the survey when the interviewers (who worked in pairs for security and logistic reasons) invited them to participate. Being interviewed in the own apartments decreases opportunity costs (thus increasing the participation rate) and to some extent prevents selection-bias (that could exist when volunteers are coming to the lab). Although this procedure does not completely eliminate self-selection, it seems rather clear that the reduction in opportunity costs related to being interviewed at home reduces possible selection-biases as well (see [37] for further arguments and analyses on this issue). Moreover, participants did not self-select into a monetarily-incentivised experiment but into a “study” (see below). In order to control for selection-bias within households, only the individual who opened the door was allowed to participate. Lastly, the data collection process was well distributed across both daytime and weekday. This sampling procedure resulted in a representative sample of the city's adult population in terms of age and gender ([37]).

### Protocol and the experimental games

The interviewers were senior University students enrolled in a course on “Field Experiments”. Their performance was linked to their final grade in the course and carefully monitored by the main researchers in real time by means of a web-based system and follow-up calls to randomly selected participants in order to ensure the reliability of the data collected. The interviewers introduced themselves to the potential participants and explained that they were carrying out a study for the University of Granada. Upon explicit oral agreement to participate, the participants were informed that the data would be used for scientific purposes only and under conditions of anonymity, according to the Spanish Law on Data Protection. One interviewer read the questions clearly, while the other noted down the answers (to the socio-economic questions). The duration of the survey-experiment averaged 40 minutes and 835 observations were finally obtained.

In the first part, extensive socio-economic information of the participants was collected. In the second part, participants played both roles of three paradigmatic games of research on social preferences, namely the dictator game (DG), the ultimatum game (UG) and the trust game (TG). Thus, each participant made five decisions, since the second player in the DG is totally passive. At the beginning of the second part, the participants received some general information about the nature of experimental economic games according to standard procedures. In particular, participants were informed that:

*i*) The five decisions involved real monetary payoffs, coming from a national research project endowed with a specific budget for this purpose;

*ii*) The monetary outcome would depend on the participant's decision, or on both her/his own and another randomly matched participant's decision, whose identity would forever remain anonymous;

*iii*) One of every ten participants would be randomly selected to be paid, and the exact payoff would be determined by a randomly selected decision (role/game);

*iv*) Matching and payment would be implemented within the next few days;

*iv*) The procedures ensure absolute double-blinded anonymity by using a decision sheet, which participants would place in the provided envelope and then seal. Thus, their decisions would remain forever unknown to: the interviewers, the researchers, and the randomly matched participant.

Once the general instructions had been explained, the interviewer read the details for each experimental decision separately. After every instruction set, participants were asked to write down their decisions privately and proceed to the next task. To control for possible order effects on decisions, the order both between and within games was randomised across participants, resulting in 24 different orders (always setting aside the two decisions of the same game). On average, the eighty subjects who were randomly selected for real payment earned €9.60 (min €0; max €40).

### Variables of interest and basic statistics

The dataset is very rich and facilitates the use of a large battery of controls. After the exclusion of the 69 observations with missing values in any of the variables used, we arrived at a sample size of 766 individuals (although 10 extra observations are excluded for the analysis of religious participation within Catholics).

Experimental design and variables: We have five basic measurements based on subjects' behaviour in the experimental games, each reflecting a dimension of social behaviour: genuine altruism, strategic altruism, sense of fairness, trust, and positive reciprocity. The derivation of these elements is described below:

In the DG, subjects had to split a “pie” of €20 between themselves and an anonymous participant. Subjects decided which share of the €20, in €2 increments, they wanted to transfer to the other participant. Hence, this variable facilitates the observation of genuine altruism/generosity.

In the case of the UG, proposers made an offer (also from a pie of €20) to the responder, but implementation was upon acceptance of the offer by the randomly matched responder. In case of rejection neither participant earned anything. For the role of the responder we used the strategy method, in which subjects have to state their willingness to accept or reject each of the proposals. Since low offers in the UG might be rejected, we consider proposers' generous offers as strategic altruism. The subjects' minimum acceptable offer (MAO) as responders in the UG – that is, the minimum amount of money that the subject would accept – reflects a sense of self-centred fairness (negative reciprocity against unfair treatment or aversion to inequality, at least to disadvantageous inequality).

In the TG (a binary version created in [50]), the trustor (1st mover) had to decide whether to pass €10 or €0 euros to the trustee (2nd mover). In case of passing nothing, the trustor earned €10 and the trustee nothing. If she/he passed the €10, the trustee would receive €40 (the amount of money was quadrupled). In the second step: the trustee, conditional on the trustor having passed the money, had to decide whether to send back €22, and keep €18 for himself, or keep all €40 without sending anything back, in which case the trustor would not earn anything. Hence, a trustor passing the money in this binary TG reflects confidence in the trustworthiness of the trustee, while the trustee returning a positive amount of money indicates positive reciprocity since she/he could keep the whole pie.

Religious dimensions: The first section of the survey includes questions on the following aspects of religiosity (relative frequencies of responses in parentheses):

Item 15 relates to religious denomination/beliefs *“As far as your religious denomination/beliefs are concerned, do you classify yourself as: No religion (31.3%, NR hereafter), Catholic (61.6%), Muslim (2%), Evangelical (0.8%), other religion (4.3%)”.*

Item 15.1 focuses on frequency of attendance of church (place of worship) services (relative frequencies among Catholics, in parentheses). “*How often do you go to church (place of worship)? Never (40.5%), less than once a month (26.6%), once in a month (14.1%), once in a week (16.7%), every day (2.2%)*”.

Items 16 and 16.1 relate to changes in the religious denomination: *“Have you ever changed your religious denomination/beliefs? Yes (16.2%), No (83.8%)”.*

Individuals who changed denomination were then asked: *“Before changing your denomination/beliefs, you identified your denomination/beliefs as: No religion (0%), Catholic (98.3%), Muslim (0%), Evangelical (0%), other religion (1.7%)”.*

The combination of information derived from questions 15 and 16 enables the calculation of the share of subjects who were (raised as) Catholics and currently claim to have “no religion” (NR). Indeed, this group comprises 12.3% of the sample, which also means that the vast majority (75.8%) of those who switched to another denomination were raised as Catholics and are now affiliated with “no religion”. This is another indication of secularisation in Spain (see also [45])

Definition of socio-economic control variables and descriptive statistics: Table 3 presents descriptive statistics (min, max, mean and SD) of the main variables of interest of this study. Block “a” relates to controls, block “b” to religious dimensions, and block “c” to experimental variables.

**Table 3:** Descriptive statistics.

*Variable*	*min*	*max*	*mean*	*SD*
*a: Controls*				
*age*	16	89	37.677	17.098
male*	0	1	0.463	0.499
*household income*	0	9	3.828	2.413
*education*	0	8	5.065	2.258
unemployed*	0	1	0.472	0.500
married*	0	1	0.365	0.482
divorced*	0	1	0.040	0.197
widowed*	0	1	0.043	0.203
cohabiting*	0	1	0.038	0.191
*impatience*	0	11	7.930	3.008
risk 1*	0	1	0.137	0.344
risk 2*	0	1	0.334	0.472
risk 3*	0	1	0.090	0.286
*cognitive skills*	0	5	2.522	1.318
*many immigr*	1	7	4.639	2.181
big public sector*	0	1	0.619	0.486
*b: Religiosity*				
Catholic*	0	1	0.616	0.487
No religion*	0	1	0.313	0.464
Active Catholic*^∧^	0	1	0.322	0.468
NR-before Cath*^†^	0	1	0.392	0.489
*c: Experimental Games*				
*DG offer*	0	20	7.833	4.285
*UG offer*	0	20	9.296	2.982
*UG MAO*	0	10	6.980	3.587
Trustor*	0	1	0.708	0.455
Trustee*	0	1	0.711	0.454

Legend: *dummy variable, ^∧^only among Catholics,^†^only among non-believers.

The definitions of control variables that are not self-explanatory are the following: *Household income* refers to self-reported household monthly income and consists of 10 categories corresponding to €0-€4,500 (in €500 increments); *Education* refers to the subject's educational level and has 9 categories from “did not study at all” to “a graduate university degree”. *Cohabiting* takes on the value of one if the subject declares living with a partner not within wedlock, and zero otherwise.

*Impatience* corresponds to the number of impatient choices the subject made in an inter-temporal choice task and captures preference for sooner-smaller rewards over larger but more delayed rewards (see [40] for further details on this survey-based discounting task). For eliciting impatience, hypothetical rewards were used due to logistical reasons and because previous evidence has shown that the use of real (vs. hypothetical) incentives does not significantly change the distribution of individual inter-temporal choices (see, e.g., [51], [52]). The measure of impatience is included as a control since the payments of the experiment were delayed, and it has been found to affect behaviour in strategic social interactions ([40], [53], [54]).

*Risk 1*, *risk 2* and *risk 3* refer to the subject's attitudes toward financial risk and are dummy variables where 1 means that the subject chose the risky option, and 0 if chose the non-risky option. As for the impatience task, decisions on risk-taking were made over hypothetical monetary incentives. Here, however, it is fair to think that the use of hypothetical instead of real gambles might have influenced subjects' choices (see for instance [55]). Risk attitudes are controlled for since payments were probabilistic and both the UG and the TG involve some strategic risk. The risk questions are the following:

*Risk 1*: 1 if option b, 0 if option a in the question: “*We flip a coin. Choose one of the following options: a. Take 1.000 Euros no matter if it is heads or tails; b. Take 2.000 Euros if it is heads and nothing if it is tails*”.

*Risk 2*: 1 if option a, 0 if option b in the question: “*Choose one of the following options: a. Take a lottery ticket with 80% chance of winning 45 Euros and 20% chance of winning nothing; b. Take 30 Euros*”.

*Risk 3*: 1 if ‘Yes’, 0 if ‘No’ in the question: “*Would you accept the following deal? We flip a coin. If it is heads you win 1,500 Euros and if it is tails you lose 1,000 Euros*”.

Note that *risk 1* captures “risk-loving” in the domain of gains when both the risky and the non-risky option have the same expected value. *Risk 2* captures risk-loving in the gains domain as well, but in a question where the risky option yields a higher expected value than the non-risky one. Finally, *risk 3* captures risk loving when the risky option involves possible losses.

*Cogn skills* refers to cognitive skills measured by the number of correct answers in a five-question mathematical test. Two additional controls are included as proxies for political orientation, as religious adherence has been associated with different political preferences, such as racism and conservative attitudes ([21]). *Many immigr* captures the degree of agreement (on a seven-point Likert scale) with the statement “there are too many immigrants in Spain”; *big public sector* is a dummy variable that takes on the value of one if the subject answers positively the question “Do you think that the public sector in Spain is too large?”.

The religiosity-related variables of block b are the following: *Active* = 1 if the respondent reports that she/he attends church services once a month or more, and  = 0 if attendance is less frequent than once a month; *NR-before Cath* = 1 if the respondent changed her/his religious denomination from Catholic to no-religion ( = 0 otherwise).

Finally, the experimental variables: *trustor = 1* if the subject passed the money to the trustee when in the role of trustor in the TG, and  = 0 if she/he did not; while *trustee* = 1 if the subject reciprocated the trustor's trust, and  = 0 otherwise.

### Ethics statement

All participants in the experiments reported in the manuscript were informed: first, about the Spanish Law 15/1999 preserving anonymity and confidentiality; second, about the content of the experiment (and the potential monetary earnings) prior to participating. Identical instructions were read aloud by the interviewers. Since literacy was not a requirement to participate (this was necessary to obtain a representative sample) we could not ask participants to read and sign the IC. Oral informed consent was obtained from all participants included in this paper. Only those who accepted completed the experiment. Those who did not accepted did not continue the experiment.

As is standard in Economic Experiment no deception was used. Anonymity was always preserved (in agreement with Spanish Law 15/1999 on Personal Data Protection) by randomly assigning a numerical code to identify the participants in the system. No association was ever made between their real names/addresses and the results. As is standard in socio-economic experiments, no ethic concerns are involved other than preserving the anonymity of participants.

This procedure (including the consent process) was checked and approved by the Vice-dean of Research of the School of Economics of the University of Granada; the institution hosting the experiments. At that time not any official IRB committee was established at the School of Economics.

## Supporting Information

File S1 Contains the following files: **Table S1**. Active Catholics (attend. ≥ once a month) vs. Non-Active Catholics (< once month). **Table S2**. NRs who were raised as Catholics versus “all-life” NRs.
(DOCX)
