# Supplementary material for: Religious Pro-Sociality? Experimental Evidence from a Sample of 766 Spaniards
Source: PLoS One. 2014 Aug 12;9(8):e104685. doi: 10.1371/journal.pone.0104685 (PMC4130547; doi:10.1371/journal.pone.0104685)
Supplement: File S1 — Contains the following files: Table S1. Active Catholics (attend. ≥ once a month) vs. Non-Active Catholics (< once month). Table S2. NRs who were raised as Catholics versus “all-life” NRs. (DOCX) [file pone.0104685.s001.docx]

**Religious pro-sociality?**

**Experimental evidence from a sample of 766 Spaniards**

**Supplementary Tables**

(Brañas-Garza, P.; Espín, A.M.; Neuman, S.)

**Table S1:** Active Catholics (attend. ≥ once a month) vs. Non-Active Catholics (< once month)

|  | DG offer | UG offer | UG MAO | Trustor | Trustee |
| --- | --- | --- | --- | --- | --- |
|  | (1) | (2) | (3) | (4) | (5) |
| *Active* | 1.028* | 0.163 | -0.851** | 0.175 | -0.103 |
|  | (0.542) | (0.292) | (0.387) | (0.159) | (0.140) |
| *age* | 0.039 | 0.045 | 0.083 | 0.027 | 0.021 |
|  | (0.086) | (0.059) | (0.057) | (0.029) | (0.025) |
| *age sq.* | -0.000 | -0.000 | -0.001 | -0.000 | -0.000 |
|  | (0.001) | (0.001) | (0.001) | (0.000) | (0.000) |
| *male* | -0.341 | -0.175 | -0.033 | -0.009 | 0.209 |
|  | (0.477) | (0.242) | (0.306) | (0.138) | (0.138) |
| *house income* | -0.344*** | -0.054 | 0.139* | -0.032 | -0.024 |
|  | (0.113) | (0.078) | (0.080) | (0.032) | (0.035) |
| *education* | 0.080 | 0.120 | -0.107 | 0.018 | -0.025 |
|  | (0.125) | (0.085) | (0.077) | (0.035) | (0.036) |
| *unemployed* | -1.323** | -0.082 | 0.071 | 0.063 | 0.012 |
|  | (0.531) | (0.305) | (0.361) | (0.136) | (0.176) |
| *married* | 0.265 | -0.701 | -0.857 | 0.181 | 0.081 |
|  | (0.766) | (0.493) | (0.536) | (0.226) | (0.250) |
| *divorced* | 1.396 | -0.222 | -0.072 | -0.019 | 0.176 |
|  | (1.037) | (0.812) | (0.886) | (0.362) | (0.378) |
| *widowed* | -0.498 | -0.725 | -0.008 | 0.230 | 0.137 |
|  | (1.129) | (0.711) | (0.857) | (0.365) | (0.468) |
| *cohabiting* | -2.306 | -2.374* | -0.018 | 0.018 | -0.636 |
|  | (2.469) | (1.435) | (1.359) | (0.385) | (0.503) |
| *impatience* | -0.038 | -0.058 | 0.026 | 0.020 | 0.023 |
|  | (0.085) | (0.049) | (0.053) | (0.022) | (0.027) |
| *risk 1* | -0.237 | -0.139 | 1.023** | -0.102 | -0.568*** |
|  | (0.596) | (0.413) | (0.472) | (0.188) | (0.219) |
| *risk 2* | 0.446 | -0.281 | 0.100 | 0.251* | -0.099 |
|  | (0.490) | (0.356) | (0.400) | (0.141) | (0.132) |
| *risk 3* | 1.945*** | 1.105** | -0.946 | 0.901*** | 0.453* |
|  | (0.622) | (0.478) | (0.642) | (0.263) | (0.236) |
| *cogn skills* | -0.108 | -0.069 | 0.410*** | -0.097* | 0.027 |
|  | (0.205) | (0.110) | (0.143) | (0.059) | (0.055) |
| *manyimmigr* | -0.139 | -0.099 | 0.009 | -0.096** | -0.082** |
|  | (0.129) | (0.084) | (0.075) | (0.040) | (0.041) |
| *big public sect.* | 0.158 | -0.230 | -0.087 | -0.177 | 0.241 |
|  | (0.459) | (0.344) | (0.333) | (0.157) | (0.160) |
| *Constant* | 9.780*** | 9.861*** | 4.422*** | 0.994 | 0.241 |
|  | (2.842) | (1.316) | (1.581) | (0.898) | (0.809) |
| *LR* | 3.253*** | 1.333 | 3.107*** | 69.559*** | 110.065*** |
| *ll* | -1223.316 | -1145.235 | -1199.287 | -256.395 | -241.397 |
| *N* | 462 | 462 | 462 | 462 | 462 |

Notes: Dependent variables are displayed on top of the columns. Tobit estimates for models (1) and (2), OLS for model (3) and Probit for models (4) and (5). Robust SEs clustered by interviewer are presented (in parentheses). All regressions control for order effects. Ten observations had missing values for church attendance and were dropped. * p<0.1, ** p<0.05, *** p<0.01.

**Table S2:** NRs who were raised as Catholics versus “all-life” NRs

|  | DG offer | UG offer | UGMAO | Trustor | Trustee |
| --- | --- | --- | --- | --- | --- |
|  | (1) | (2) | (3) | (4) | (5) |
| *NR-before Cath* | 0.223 | 0.037 | -0.037 | -0.252 | 0.256 |
|  | (0.734) | (0.393) | (0.424) | (0.212) | (0.237) |
| *age* | -0.001 | -0.004 | 0.045 | 0.027 | -0.023 |
|  | (0.177) | (0.082) | (0.101) | (0.038) | (0.060) |
| *age sq.* | 0.001 | -0.000 | 0.000 | -0.000 | 0.000 |
|  | (0.002) | (0.001) | (0.001) | (0.000) | (0.001) |
| *male* | -0.838 | -0.205 | -0.564 | -0.176 | 0.054 |
|  | (0.738) | (0.397) | (0.547) | (0.201) | (0.218) |
| *house income* | -0.008 | 0.069 | 0.150 | -0.022 | -0.037 |
|  | (0.170) | (0.087) | (0.116) | (0.048) | (0.043) |
| *education* | -0.023 | 0.087 | -0.084 | -0.044 | 0.104** |
|  | (0.199) | (0.126) | (0.136) | (0.053) | (0.051) |
| *unemployed* | 0.899 | -0.091 | 0.984 | 0.314 | -0.411** |
|  | (0.943) | (0.390) | (0.609) | (0.236) | (0.207) |
| *married* | 0.752 | 0.706 | -2.376** | 0.335 | -0.022 |
|  | (1.220) | (0.776) | (1.176) | (0.367) | (0.381) |
| *divorced* | 1.624 | -1.126 | -3.403** | 0.171 |  |
|  | (1.466) | (1.699) | (1.648) | (0.883) |  |
| *widowed* | -6.162 | 1.978 | 0.285 | 0.338 |  |
|  | (4.058) | (1.248) | (1.878) | (0.791) |  |
| *cohabiting* | 0.292 | -0.319 | -0.512 | 0.840* | -0.340 |
|  | (1.369) | (0.439) | (0.772) | (0.467) | (0.424) |
| *impatience* | -0.219* | -0.123** | 0.193** | -0.005 | -0.034 |
|  | (0.120) | (0.057) | (0.093) | (0.030) | (0.036) |
| *risk 1* | -0.243 | 0.141 | 1.878*** | -0.279 | -0.394 |
|  | (1.177) | (0.579) | (0.589) | (0.274) | (0.280) |
| *risk 2* | 0.958 | 0.067 | -0.198 | 0.230 | -0.294 |
|  | (0.896) | (0.420) | (0.504) | (0.217) | (0.231) |
| *risk 3* | 1.239 | 0.175 | -0.119 | 1.010* | 1.103* |
|  | (1.106) | (0.477) | (0.836) | (0.565) | (0.564) |
| *cogn skills* | -0.292 | 0.000 | -0.238 | 0.353*** | 0.251** |
|  | (0.394) | (0.189) | (0.193) | (0.090) | (0.107) |
| *manyimmigr* | -0.436*** | -0.066 | -0.020 | -0.048 | 0.009 |
|  | (0.159) | (0.104) | (0.121) | (0.047) | (0.047) |
| *big public sec* | 0.929 | 0.202 | 0.801* | 0.383** | -0.191 |
|  | (0.710) | (0.384) | (0.426) | (0.188) | (0.213) |
| *Constant* | 9.523** | 5.756 | 6.767*** | -2.014** | 0.945 |
|  | (4.481) | (3.532) | (2.460) | (0.849) | (1.188) |
| *LR* | 3.9278*** | 2.574*** | 5.310*** | 93.075*** | 71.413*** |
| *ll* | -624.169 | -566.919 | -623.767 | -118.376 | -110.599 |
| *N* | 240 | 240 | 240 | 240 | 229 |

Notes: Dependent variables are displayed on top of the columns. Tobit estimates for models (1) and (2), OLS for model (3) and Probit for models (4) and (5). Robust SEs clustered by interviewer are presented (in parentheses). *widower*=1 and *divorced*=1 predict success perfectly in model (6), thus the two variables are dropped and 11 observations not used. All regressions control for order effects. * p<0.1, ** p<0.05, *** p<0.01.
